# β-Nicotinamide mononucleotide improves chilled ram sperm quality *in vitro* by reducing oxidative stress damage

**DOI:** 10.5713/ab.23.0379

**Published:** 2024-04-01

**Authors:** Zhendong Zhu, Haolong Zhao, Qitai Yang, Yajing Li, Ruyuan Wang, Adedeji Olufemi Adetunji, Lingjiang Min

**Affiliations:** 1 College of Animal Science and Technology, Qingdao Agricultural University, Qingdao 266109, China; 2 Department of Agriculture, University of Arkansas at Pine Bluff, Pine Bluff, AR 71601, USA

**Keywords:** β-Nicotinamide Mononucleotide, Oxidative Stress, Ram, Sperm, Storage

## Abstract

**Objective:**

The present study aimed to investigate the effect of β-nicotinamide mononucleotide (NMN) supplementation on ram sperm quality during storage at 4°C *in vitro*.

**Methods:**

Tris-citric acid-glucose solution containing different doses of NMN (0, 30, 60, 90, and 120 μM) was used to dilute semen collected from rams and it was stored at 4°C. Sperm motility, plasma membrane integrity as well as acrosome integrity were evaluated at 0, 24, and 48 h time points after storage at 4°C. In addition, sperm mitochondrial activity, lipid peroxidation (LPO), malondialdehyde (MDA) content, reactive oxygen species (ROS) content, glutathione (GSH) content, superoxide dismutase (SOD) activity, and apoptosis were measured at 48 h time point after storage at 4°C.

**Results:**

Results demonstrate that the values obtained for sperm motility, acrosome integrity, and plasma membrane integrity in the NMN treatments were significantly higher than control (p<0.05). The addition of 60 μM NMN significantly improved ram sperm mitochondrial activity and reduced LPO, MDA content, and ROS content compared to control (p<0.05). Interestingly, sperm GSH content and SOD activity for the 60 μM NMN treatment were much higher than those observed for control. NMN treatment also decreased the level of Cleaved-Caspase 3, Cleaved-Caspase 9, and Bax while increasing Bcl-2 level in sperm at 48 h time point after storage at 4°C.

**Conclusion:**

Ram sperm quality can be maintained during storage at 4°C with the addition of NMN at 60 μM to the semen extender. NMN also reduces oxidative stress and apoptosis. Overall, these findings suggest that NMN is efficient in improving the viability of ram sperm during storage at 4°C *in vitro*.

## INTRODUCTION

Artificial insemination (AI) is an assisted reproductive technology that facilitates the genetic improvement of livestock by increasing the use of superior males with highly productive traits [1]. It is also important for genetic improvement in sheep production and breeding activities [1]. However, the type of AI techniques to be used in sheep depends on the semen deposition method to be adopted, and the options available are vaginal, cervical, and intracervical deposition methods [2]. Likewise, ram semen used for AI can be fresh, chilled, or frozen. Generally, the intravaginal method is used when working with chilled semen because it is simple and cost-effective for commercial flocks [3]. Previous studies have reported lower pregnancy rates with chilled semen compared to natural mating [4]. In addition, sperm motility and viability rates decreased during storage at the rate of 60%, 52%, 30%, and 18% for 0, 24, 48, and 72 h, respectively [5]. Sperm quality is an important factor that affects pregnancy rate, however, storing sperm in chilled conditions causes changes in sperm membrane function and structure, and shortens its lifespan [6]. Also, compared to fresh semen, the progressive motility of chilled semen in the female reproductive tract is slower [7]. Therefore, it is imperative to explore chilled semen quality improvement techniques to increase the success of AI in sheep.

Reactive oxygen species (ROS) is generated by the nicotinamide adenine dinucleotide phosphate (NADPH) oxidase system and the NADPH-dependent redox reaction in the sperm membrane and sperm, respectively [8]. Most ROS produced in sperm is a result of electron leakage from the mitochondria which occurs during storage under unphysiological conditions *in vitro* [9]. Consequently, an increase in the number of dysfunctional sperm in the semen during storage *in vitro* at 4°C is the major cause of elevated ROS levels. Antioxidants present in sperm usually quench excessive ROS to prevent damage to sperm cells. However, an imbalance between sperm antioxidant capa city and the amount of ROS generated leads to ROS accumulation. This occurrence adversely affects sperm motility and fertility through lipid peroxidation (LPO), DNA fragmentation, and apoptosis [10]. Previous studies have reported ROS accumulation during ram sperm storage at 4°C [11]. In addition, the viability of ram sperm decreases during storage at 0°C to 15°C after 24 h as a result of ROS-induced disruption of the biological function of ram sperm. Moreover, researchers have explored the use of compounds with inherent antioxidant properties, such as resveratrol [12], alginate oligosaccharide [13] in chilled ram sperm storage. To this end, we investigated the use of β-nicotinamide mononucleotide (NMN) in enhancing the quality of chilled ram sperm after storage, leveraging its antioxidant capacity.

NMN is a bioactive nucleotide and a natural antioxidant synthesized by NAMPT, an enzyme involved in nicotinamide adenine dinucleotide (NAD^+^) production in mammals [14]. NMN widely exists in vegetables, fruits, and meat [15]. Studies have shown that NMN can reduce ROS, LPO, and sperm DNA damage by increasing Sirtuin 1 (SIRT1) activity [16]. It has also been shown to reverse NAD^+^ deficiency-induced anomaly in DNA repair, cell survival, mitochondrial homeostasis, and ROS production [17]. However, available information about the effect of NMN on ram sperm storage is limited. This study aimed to investigate the effect of NMN supplementation on ram sperm quality during storage at 4°C.

## MATERIALS AND METHODS

### Animal care

All animals and experimental procedures were approved by the Qingdao Agriculture University Institutional Animal Care and Use Committee (QAU1121010).

### Chemicals and extenders

Unless specifically indicated, the chemicals and reagents used in this study were obtained from Sigma-Aldrich (Shanghai, China). Tris-citric acid-glucose (TCG) solution, consisting of 69 mM glucose, 83 mM citric acid, 250 mM Tris, 100 μg/mL polymyxin B, 1,000 IU/mL penicillin G sodium salt (Solarbio, Beijing, China), and 1 mg/mL streptomycin sulfate was used as an extender (Solarbio, China).

### Animal semen collection

Semen was collected from five healthy and fertile rams (Small-Tailed Han sheep; about 2 years of age) from each ram twice a week for 4 successive weeks using an artificial vagina in December 2021 at the Hongde livestock farm (Shouguang, China). A total of 40 ejaculates were obtained, and each ejaculate was incubated at 37°C. Ejaculated semen motility was estimated using computer-assisted sperm analysis (CASA) and only samples with over 80% motility were used. Also, sperm concentration was assessed using a hemocytometer, and only semen with a density exceeding 2×10^9^ sperm/mL was used in this study. The ejaculated semen from the rams were pooled and split into 5 parts for subsequent analysis.

### Semen processing

Semen collected were diluted with TCG medium containing different concentrations of NMN (0, 30, 60, 90, and 120 μM) at a final concentration of 400×10^6^ sperm/mL. The samples were cooled in a cold cabinet from 37°C to 4°C. After that, they were stored at 4°C. Sperm motility parameters, plasma membrane integrity, and acrosome integrity were evaluated at 0, 24, and 48 h time points and stored at 4°C. Sperm mitochondrial activity, LPO, malondialdehyde (MDA) content, glutathione (GSH) content, ROS content, superoxide dismutase (SOD) activity, and apoptosis were evaluated after 48 h of storage at 4°C.

### Assessment of sperm motility

Computer-assisted sperm analysis (SCA 20-06-01; Goldcyto, Barcelona, Spain) was performed. For detection, images were acquired using a digital camera (acA780-75gc; Basler, Allensburg, Germany) and a negative phase contrast microscope at 100× magnification, set to a standard parameter of 25 frames/s. According to our previous study [18], after preheating the analyzer, semen sample aliquots of 5 μL were added to the Makler chamber. Sperm motility was assessed in three randomly selected areas using CASA, and a total of 500 sperm were evaluated. The percentage of sperm moving at a path speed of 12 μm/s was defined as total sperm motility. Forward movement denotes the percentage of sperm moving in a straight line at a path velocity of 45 μm/s for more than 80% of the time.

### Evaluation of sperm acrosome integrity and plasma membrane integrity

According to Dziekońska et al [19], sperm acrosome integrity and plasma membrane integrity were detected by fluorescein isothiocyanate-peanut lectin (FITC-PNA, L-7381; Sigma-Aldrich, China) and LIVE/DEAD sperm viability test kit (L-7011; Thermo Fisher, Shanghai, China), respectively. To evaluate sperm acrosome integrity, sperm samples were incubated with fluorescein isothiocyanate-peanut lectin solution (100 μg/mL) and a solution of 2.4 mM propidium iodide (PI) at 37°C for 5 min in the dark. For plasma membrane integrity detection, sperm samples were incubated with 100 nM SYBR-14 working solution and 2.4 mM PI solution in the dark for 10 min. Moreover, the stained sperm were photographed after monitoring using a fluorescence microscope at 400× magnification to evaluate acrosome integrity and plasma membrane integrity (emitting green fluorescence at 516 nm and red fluorescence at 617 nm) as previously described by Zhu et al [20]. The result for acrosome integrity denotes the percentage of sperm with intact acrosomal membrane integrity and plasma membrane integrity (not stained by FITC-PNA/PI min the sperm head; FITC-PNA^−^/PI^−^ and SYBR-14^+^/PI^−^, respectively). A total of 300 sperm were evaluated. Moreover, the analyses were performed in triplicate.

### Evaluation of mitochondrial activity

According to Dziekońska et al [19], JC-1 fluorescent probe (JC-1) and PI staining were carried out to evaluate sperm mitochondrial activity. Sperm samples were incubated for 15 min at 37°C in the dark with 1 μL JC-1 (1 mg JC-1/mL anhydrous dimethyl sulfoxide). Then, it was incubated in the dark with 2 μM PI for a total of 5 min at 37°C. The stained sample was evaluated with a fluorescence microscope at 400× magnification. Sperm with an orange fluorescence in the midpiece were considered viable with high mitochondrial activity (JC-1^+^/PI^−^). Meanwhile, sperm consisting of green fluorescence in the midpiece while lacking fluorescence in the head were categorized as viable sperm with low mitochondrial membrane potential (MMP). Also, the results were presented as the percentage of viable sperm with high mitochondrial activity. In addition, a ratio of the number of sperm with high mitochondrial activity compared to the total sperm count was obtained. A total of 300 sperm were evaluated. The analyses were performed in triplicate.

### Measurement of sperm malondialdehyde content

MDA is one of the final products of polyunsaturated fatty acids (PUFA) peroxidation in cells, which is regarded as a marker of oxidative stress. According to Zhang et al [18], a commercial MDA assay kit (S0131S; Beyotime Institute of Biotechnology, Shanghai, China) was used to measure MDA content. Sperm stored at 4°C were lysed by sonication (20 kHz, 300 W, operating at 50%, 3 min for 10 s on and 5 s off) on ice. Then the sample was mixed with the pre-prepared reaction buffer reagent and boiled for 40 min. After cooling, the sample was centrifuged, and the supernatant was collected. Furthermore, absorbance was taken at 532 nm using a microplate reader (TECAN; Infinite M Nano, Männedorf, Switzerland). Meanwhile, BCA protein quantification kit (E112-01; Vazyme, Nanjing, China) was used to detect the sperm protein level. The analyses were performed in triplicate.

### Detection of lipid peroxidation

LPO occurs under conditions where ROS react with vulnerable lipids on the cell membrane, leading to degrading sperm quality. According to Li et al [21], a LPO assay kit (A160-1; Nanjing Jiancheng Bioengineering Institute, Wuhan, China) was used to measure LPO content. In brief, saline was added to the sperm samples and homogenized in an ice water bath. Thereafter, the sample was centrifuged and mixed with the prepared buffer. Then, absorbance was taken at a wavelength of 586 nm using a microplate reader (TECAN; Infinite M Nano, Switzerland). The analyses were performed in triplicate.

### Detection of glutathione content

GSH plays a key role in maintaining cellular redox homeostasis. According to Zhu et al [20], a commercial GSH assay kit (A061-1, Nanjing Jiancheng Bioengineering Institute, China) was used to measure sperm GSH content. For the determination of total glutathione (T-GSH), the sperm samples were homogenized and centrifugated at 3,500 rpm/min and the supernatant was collected. Then, reagents were added and mixed with the supernatant based on the manufacturer’s instructions. The absorbance (A1) was measured at 532 nm using a microplate reader after waiting for 30 s, while absorbance (A2) was measured after standing at room temperature for 10 min. To detect oxidized glutathione (GSSG), reagents were added, mixed with the supernatant, and incubated at 37°C for 30 min according to the manufacturer’s instructions. Thereafter, reagents were added and mixed again, and absorbance (A1) and absorbance (A2) were read at 450 nm based on manufacturer instructions. GSH content was determined using the formula: GSH = T-GSH-2×GSSG. The analyses were performed in triplicate.

### Measurement of superoxide dismutase activity

SOD enzymes are considered the first line of defense against ROS. According to Zhang et al [22], SOD activity was measured by a total SOD detection kit (A001-3-2; Nanjing Jiancheng Bioengineering Institute, China). Sperm cells were crushed by frozen ultrasound (300 W, every 5 s, 4 times at an interval of 30 s), and the supernatant was used for SOD activity detection. The enzyme working solution followed by the substrate solution was added to the supernatant and incubated for 20 min at 37°C. Thereafter, absorbance was taken with the aid of a microplate reader at 450 nm (TECAN; Infinite M Nano, Switzerland). SOD activity (U/mg prot) = SOD inhibition rate/(50%×12)/protein concentration. The analyses were performed in triplicate.

### Measurement of sperm reactive oxygen species

According to Zhu et al [23], a ROS detection kit (M36008; Thermo Fisher Scientific, China) was used to determine the content of ROS. Sperm samples were centrifuged and resuspended with 200 μL working solution. The cells were incubated for 15 min in the dark at 37°C. Thereafter, the cells were centrifuged and washed three times with 1× phosphate buffered saline (1×PBS). Stained sperm were re-suspended in 1× PBS and evaluated by flow cytometry (FACS Aria III; BD Biosciences, Franklin Lakes, NJ, USA) using a filter with a bandwidth of 574/26 nm, and measurements denote the mean fluorescence intensity. The analyses were performed in triplicate.

### Western blotting

Total sperm protein was extracted using sodium dodecyl sulfate (SDS) sample buffer. Moreover, total proteins (20 μg) from each sample were separated on a 10% sodium dodecyl sulphate-polyacrylamide gel electrophoresis (SDS-PAGE) (E303-01; Vazyme, China), and the separated proteins were transferred to a polyvinylidene difluoride (PVDF) membrane. Nonspecific binding of PVDF membrane was blocked by tris buffered saline with tween-20 (TBST) containing 5% bovine serum albumin (BSA). Then, 1% BSA (dissolved in TBST) was used to dilute primary antibodies such as Cleaved-Caspase 3 (A22869, Rabbit monoclonal; AB clone, Wuhan, China), Cleaved-Caspase 9 (A22672, Rabbit monoclonal; AB clone, China), Bax (A0207; AB clone, Rabbit monoclonal, China), Bcl-2 (A0208; AB clone, Rabbit monoclonal, China), anti-α-tubulin (AC007; AB clone, Rabbit monoclonal, China) at a ratio of 1:1,000, and incubated for a total of 12 h at 4°C. Then, the PVDF membranes were placed in a TBST solution for washing. Thereafter, it was incubated with a secondary antibody (AS014, Goat anti-rabbit IgG, 1:1,000; AB Clone, China) for 1 h. ECL plus (ED0016-B; Sparkjade, Jinan, China) was added to the membrane for detection prior to developing the image with a gel imaging analyzer (Alpha; Fluor Chem Q, Shanghai, China).

### Statistical analysis

Data from three replicates were compared using one-way analysis of variance followed by Tukey’s post hoc test (Statview; Abacus Concepts, Inc., Berkeley, CA, USA). All the values in this study are presented as the mean±standard error of the mean. Moreover, treatments were considered to be statistically different from one another at p<0.05.

## RESULTS

### Addition of β-nicotinamide mononucleotide improved chilled sperm motility parameters

As shown in Table 1, when the motility patterns of sperm were analyzed through the movement trajectories generated by CASA, it was found that the addition of 60 or 90 μM NMN to the extender significantly increased (p<0.05) the sperm total motility, progressive motility, VSL (straight-line velocity), VAP (average path velocity), LIN (linearity) VCL (curvilinear velocity), and STR (straightness) at 24 h and 48 h points of storage at 4°C. Specifically, the 60 μM NMN treatment had the highest value for the parameters above. In addition, at 0 h of the NMN treatments, there are no differences (p> 0.05) in the sperm parameters among the treatments (Supplementary Table S1). Regarding ALH (lateral head) and BCF (beat-cross frequency) parameters, there were also no differences (p>0.05) among the treatments after storage at 4°C at 24 h or 48 h points.

### Addition of β-nicotinamide mononucleotide improved the sperm acrosome integrity and plasma membrane integrity

**Figure 1:**
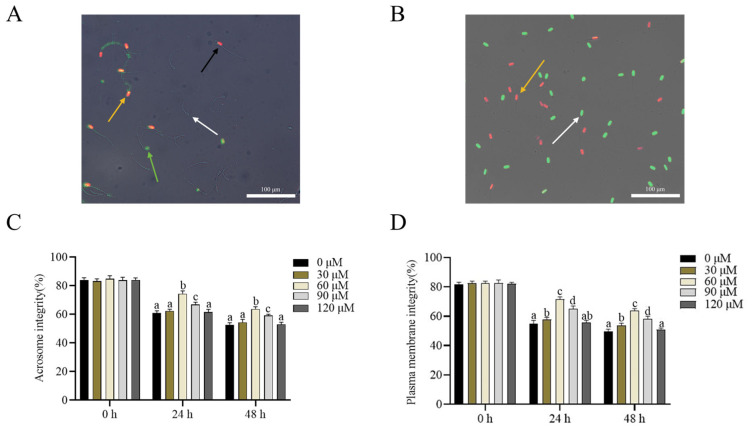
Photographs of ram sperm stained with fluorescein isothiocyanate-peanut aggluti-nin/PI (A) and LIVE/DEAD sperm viability kit (B), respectively. In Figure A, white arrow indicates viable sperm with intact acrosome, green arrow indicates viable sperm with damaged acrosome, orange arrow indicates dead sperm with damaged acrosome, and black arrow indicates dead sperm with intact acrosome. In Figure B, the white arrow indicates the sperm with intact plasma membrane, while the orange arrow indicates sperm with damaged plasma membrane. Effect of addition of different concentrations of β-nicotinamide mononucleotide to extender on acrosome integrity (C) and plasma membrane integrity (D) of ram sperm after storage at 4°C. Values are presented as mean±standard error of the mean. ^a–d^ Columns with different lowercase letters at the same time are significantly different (p<0.05), n = 5. Bars = 100 μm.

As shown in Figure 1A, four kinds of sperm were observed under the fluorescence microscope after the sperm was stained with FITC-PNA/PI: the white arrow indicates viable sperm with intact acrosome; the green arrow indicates viable sperm with damaged acrosome; the orange arrow indicates dead sperm with damaged acrosome; and the black arrow indicates dead sperm with intact acrosome. The addition of NMN to the extender greatly improved the integrity of sperm acrosomes after storage at 4°C at 24 and 48 h points (Figure 1C). The 60 μM NMN treatment group showed more intact acrosomes than the control group (Figure 1C). In addition, as shown in Figure 1B, the white arrow indicates sperm with an intact plasma membrane, while the orange arrow indicates sperm with a damaged plasma membrane. Moreover, the addition of 60 or 90 μM NMN to the extender significantly improved (p<0.05) sperm plasma membrane integrity after storage at 4°C at 24 and 48 h points. Specifically, 60 μM NMN treatment had the highest value among the treatments (Figure 1D).

### Addition of β-nicotinamide mononucleotide improved sperm mitochondrial activity

**Figure 2:**
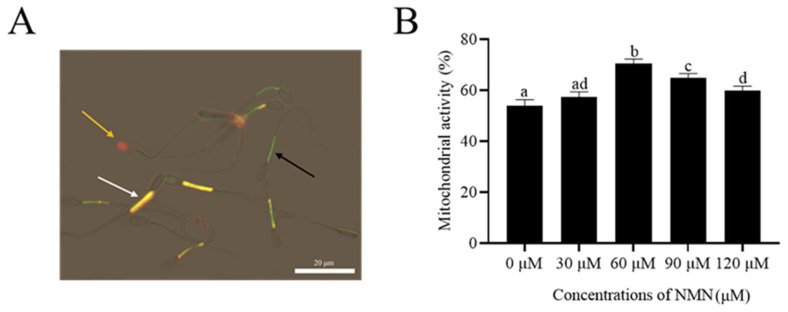
Photographs of ram sperm stained with a mixture of JC-1 fluorescent probe (JC-1) and propidium iodide (PI) (A). Sperm with an orange fluorescence in the midpiece were considered viable with high mitochondrial activity (JC-1^+^/PI^−^, white arrow), meanwhile, sperm with green fluorescence in the mid-piece and no fluorescence in the head were classified as viable sperm with low mitochondrial membrane potential (MMP) (black arrow). The orange arrow indicates dead sperm. Effect of different concentrations of β-nicotinamide mononucleotide (NMN) on mitochondrial activity after storage at 4°C (B). Values are presented as mean±standard error of the mean. ^a–d^ Columns with different lowercase letters are significantly different (p<0.05), n = 3. Bar = 20 μm.

As shown in Figure 2A, sperm with an orange fluorescence in the midpiece were considered viable with high mitochondrial activity (JC-1^+^/PI^−^; white arrow). Meanwhile, sperm with green fluorescence in the mid-piece and no fluorescence in the head were classified as viable sperm with low MMP (black arrow). The orange arrow indicates dead sperm. The addition of NMN to the extender increased (p<0.05) sperm mitochondrial activity after storage at 4°C for 48 h (Figure 2B). Moreover, both 60 and 90 μM NMN treatments had the highest value among the treatments (Figure 2B).

### β-Nicotinamide mononucleotide reduced the reactive oxygen specieslevel in sperm during the storage at 4°C

**Figure 3:**
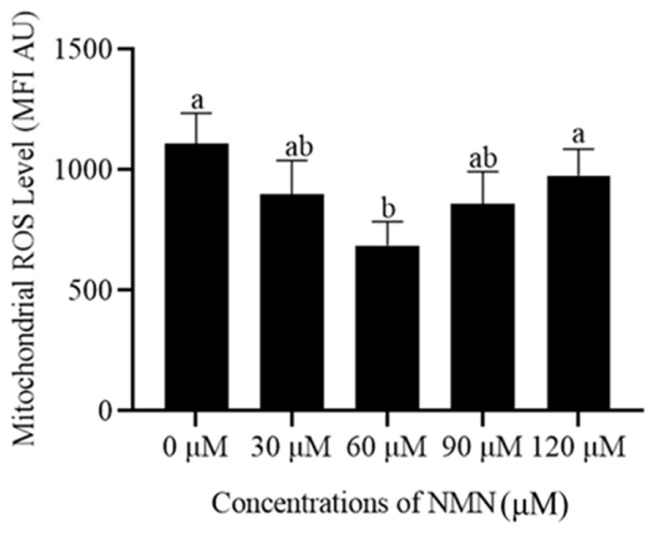
Effect of different concentrations of β-nicotinamide mononucleotide (NMN) on sperm reactive oxygen species after storage at 4°C. Values are presented as mean±standard error of the mean. ^a,b^ Columns with different lowercase letters were significantly different (p<0.05), n = 3.

As shown in Figure 3 and Supplementary Figure 1, the addition of 60 μM NMN to the extender significantly decreased (p<0.05) sperm ROS content after storage at 4°C, while the 30, 90, and 120 μM NMN treatments showed similar values of ROS content compared to the control (Figure 3).

### Addition of β-nicotinamide mononucleotide to the extender improved sperm anti-oxidative stress

**Figure 4:**
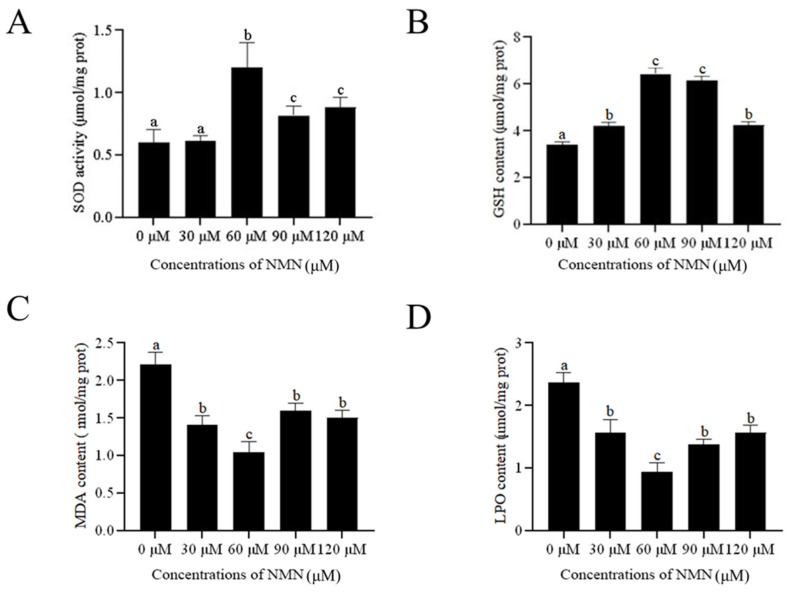
Effect of different concentrations of β-nicotinamide mononucleotide (NMN) on sperm superoxide dismutase (SOD) activity (A), glutathione (GSH) content (B), malondialdehyde (MDA) content (C) and lipid peroxidation (LPO) content (D) after storage at 4°C. Values are presented as mean±standard error of the mean. ^a–c^ Columns with different lowercase letters were significantly different (p<0.05), n = 3.

To investigate the protective effect of NMN on ram sperm stored at 4°C, the SOD activity, MDA, GSH, and LPO levels were measured. As shown in Figure 4A, SOD activity was significantly higher (p<0.05) in the NMN treatments than in the control. The highest SOD activity was observed in the 60 μM NMN treatment. However, there was no difference (p>0.05) in SOD activity between the 30 μM NMN treatment and control. In addition, the addition of NMN at concentrations between 60 to 120 μM significantly increased (p<0.05) GSH content (Figure 4B). The MDA content in NMN treatments was significantly decreased compared to the control, and the lowest MDA content was observed in the 60 μM NMN treatment (Figure 4C). Similarly, the addition of 30 to 120 μM NMN led to the reduction of sperm LPO (Figure 4D).

### Addition of β-nicotinamide mononucleotide attenuated the sperm apoptosis

**Figure 5:**
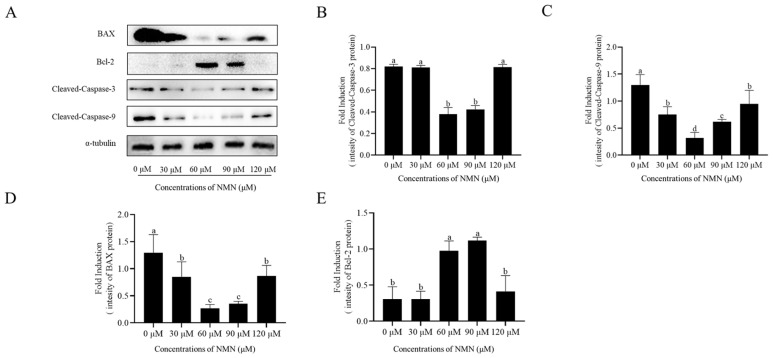
Effect of different concentrations of β-nicotinamide mononucleotide (NMN) on sperm apoptosis after storage at 4°C. Detection of the expression of Cleaved-Caspase3, Cleaved-Caspase9, BAX, and Bcl-2 by Western blot (A–E). Values are presented as mean±standard error of the mean. ^a–d^ Columns with different lowercase letters were significantly different (p<0.05), n = 3.

The expression of BAX, Cleaved-Caspase 3, and Cleaved-Caspase 9, and Bcl-2 in ram sperm after storage at 4°C was detected by western blot. The addition of 60 and 90 μM NMN significantly decreased the expression of BAX, Cleaved-Caspase 3, and Cleaved-Caspase 9 and increased the expression of Bcl-2 (Figure 5; Supplementary Figure S2), whereas sperm treated with 30 or 120 μM NMN showed no significant difference to the control.

## DISCUSSION

The addition of antioxidants to the diluted semen of cattle, horses, pigs, and sheep can significantly improve semen quality by reducing the accumulation of ROS during *in vitro* storage. NMN is a known antioxidant involved in metabolism, DNA repair, mitochondrial maintenance, and cell death [24]. In this study, the addition of NMN to the extender significantly improved the quality of ram sperm via its antioxidant capacity during liquid storage at 4°C.

Sperm motility parameters (total motility, progressive motility, VSL, VCL, VAP, STR, and LIN patterns), plasma membrane integrity, and acrosome integrity all play an essential role in sperm penetration of the zona pellucida and the completion of conception after AI [25]. In this study, sperm motility, progressive motility, VCL, VSL, VAP, and ALH parameters for 60 μM NMN treatment were significantly higher than control during storage at 4°C. Our results agree with the findings of Kiss et al [26], which shows that NMN improves mitochondrial bioenergetics. We also observed that mitochondrial activity in NMN treatments was much higher than that of control, suggesting that NMN treatment enhanced ram sperm mitochondrial activity, hereby increasing sperm motility parameters. However, the addition of 120 μM NMN did not improve sperm motility, progressive motility, VCL, VSL, VAP, and ALH parameters during the storage at 4°C. The inefficiency observed at this concentration may be because at higher doses NMN induces cytotoxicity. Unfortunately, we did not investigate this phenomenon in the present study. Youngson et al [27] showed that administering 400 mg/kg NMN to male mice fed a high-fat diet reduced sperm viability compared to the non-NMN treatment. The differences in our findings may be attributed to the disparity in NMN dosage and species. In addition, when sperm mitochondrial activity was analyzed in this study, an increase in the mitochondrial activity of NMN treatments was observed. This agrees with previous findings that NMN administration enhanced mitochondria function [28]. Generally, NAD^+^ is a cofactor that supports fundamental mitochondrial functions such as oxidative phosphorylation and many enzymatic reactions that occur in the tricarboxylic acid cycle (TCA cycle) [29]. Also, the adenylation of NMN leads to the formation of NAD^+^ by the action of nicotinamide nucleotide adenylyltransferase (NMNAT) enzyme [30]. Previous research has shown that the NMN treatment increased mitochondrial NAD^+^ levels [26]. In addition, the cellular NAD^+^ levels that determine the activity of sirtuin 3 are localized to the mitochondrial matrix [31] and they play a role in the regulation of protein acetylation [32]. Mitochondrial protein acetylation controls the functioning of enzymes involved in the TCA cycle, respiratory chain complexes, ROS degradation, and mitochondrial dynamics [33]. This suggests that NMN addition to the medium may improve sperm mitochondrial activity by increasing the sperm NAD^+^ involved in initiating sirtuin 3 activity.

Free radicals generated have a damaging effect on the PUFA of cell membranes. Because ram sperm contains a high content of PUFA in its plasma membrane, it is susceptible to oxidative stress [34]. Usually, the antioxidants (enzymatic and non-enzymatic) in sperm can reduce free radicals to maintain sperm redox homeostasis. However, during unphysiological ram sperm storage at 4°C *in vitro*, high amounts of ROS are produced. Therefore, reducing ROS levels and ROS-induced sperm damage is essential to improving the quality of sperm preserved *in vitro*. In the present study, it was observed that ROS level, LPO, and MDA content in NMN treatments were lower than those of control, suggesting that NMN treatments reduced the level of oxidative stress in ram sperm stored *in vitro* at 4°C. Our findings are consistent with a previous report that the supplementation of NMN reduces ROS production [28]. ROS accumulation causes oxidative stress conditions which lead to sperm plasma membrane and acrosome integrity damage [35]. In this study, both the acrosome integrity and sperm plasma membrane integrity increased with the addition of 60 μM NMN, interestingly, similar trends were also observed in our ROS and LPO results. Also, when ram sperm GSH content and SOD activity were evaluated, the 60 μM NMN treatment significantly increased the values of both parameters. We also observed a reduction in ROS levels with increasing GSH content and SOD activity. The increase in sperm antioxidant capacity with NMN supplementation may be due to NAD^+^ action in stimulating the activity of related enzymes [36]. This result suggests that the addition of 60 μM NMN reduces ram sperm oxidative stress during storage at 4°C.

Proteins of the B-cell lymphoma-2 denoted as Bcl-2 family are associated with the intrinsic apoptosis pathway. BAX (pro-apoptotic Bcl-2 protein) causes programmed cell death in a cell due to the initiation of the caspase cascade which occurs when the the outer mitochondrial membrane is permeabilized [37]. Afkhami-Ardakani et al [38] demonstrated that spirulina platensis was able to increase Bcl-2 expression, thereby reducing the level of apoptosis in mouse sperm. Lv et al [39] reported that the apoptosis of frozen-thawed cattle sperm was reduced by the addition of crocin which increases the Bcl-2/Bax ratio. Another key member of the caspase family is caspase-9, which is involved in apoptosis and cytokine processing. Among the downstream caspases, caspase-3 is cleaved by caspase-9 to initiate the caspase cascade [40]. Yu et al [41] indicated that LBP (Lycium barbarum polysaccharide) may reduce caspase-3 levels, thus significantly reducing cell apoptosis. Circu and Aw [42] pointed out that GSH/GSSG reduction makes protein tyrosine phosphatases open, and causes the release of apoptotic protease activator, cytochrome C, and other apoptosis-inducing factors from the mitochondria, which triggers the initiation of caspase 9 and caspase 3. In the present study, following the evaluation of ram sperm apoptosis after storage at 4°C, a decrease in Bax, Cleaved-Caspase 3, and Cleaved-Caspase 9 levels was observed in the NMN treatments. Also, NMN treatment increased the Bcl-2 level. Notably, excessive ROS generation stimulates the apoptotic process, and reducing pathological ROS levels in sperm can help prevent apoptosis.

## CONCLUSION

In conclusion, the addition of NMN to the diluent improved sperm motility, plasma membrane integrity, acrosome integrity, mitochondrial activity, and antioxidant capacity, as well it reduced sperm oxidative stress and apoptosis. NMN’s action in maintaining sperm quality during storage can be attributed to its antioxidant properties. Overall, our findings suggest that NMN can be used effectively to maintain ram sperm quality during storage at 4°C. Furthermore, the addition of 60 μM NMN improves the quality of ram sperm during storage at 4°C compared to other treatments.

## Figures and Tables

**Table 1:** Sperm motility parameters after the addition of β-nicotinamide mononucleotide to the extender as analyzed by computer-assisted sperm analysis

Items	Time	24 h					48 h				
	
	NMN	0 μM	30 μM	60 μM	90 μM	120 μM	0 μM	30 μM	60 μM	90 μM	120 μM
Total motility (%)		42.77±4.59^c^	43.71±5.09^bc^	70.51±3.61^a^	64.93±5.26^a^	49.92±4.67^b^	37.51±0.81^d^	40.82±4.48^c^	61.31±2.19^a^	53.43±4.69^b^	43.56±3.71^c^
Progressive motility (%)		33.97±5.89^c^	34.2±5.67^c^	52.45±5.64^a^	47.4±1.71^b^	34.02±1.18^c^	26.15±0.65^d^	27.72±5.29^cd^	45.55±6.75^a^	39.73±5.35^b^	30.61±1.71^c^
VCL (μm/s)		42.62±4.57^d^	49.05±2.37^c^	69.34±3.81^a^	59.41±1.67^b^	50.88±2.06^c^	31.88±4.27^d^	40.65±1.81^c^	56.11±4.96^a^	49.53±3.27^b^	39.41±4.32^c^
VSL (μm/s)		33.29±2.87^d^	38.81±1.55^c^	54.58±3.39^a^	49.63±1.02^b^	39.24±5.83^c^	27.58±0.98^d^	36.43±4.02^b^	44.75±3.68^a^	42.49±4.76^a^	31.31±3.61^c^
VAP (μm/s)		29.19±3.46^c^	36.31±1.33^b^	50.14±2.81^a^	48.69±3.94^a^	35.68±4.88^b^	23.14±2.06^c^	32.87±4.34^b^	40.54±2.68^a^	38.52±6.41^a^	33.42±3.27^b^
BCF (Hz)		7.01±0.57	6.16±0.51	4.13±0.79	8.52±0.61	5.31±0.91	5.26±0.34	4.52±0.44	4.01±0.13	5.46±0.47	4.69±1.32
ALH (μm)		3.42±0.28	3.49±0.42	4.68±0.73	4.65±0.22	4.11±0.31	2.65±0.21	3.23±0.22	4.32±0.51	4.07±0.41	3.05±0.38
STR (%)		75.24±1.11	77.76±4.36	75.18±3.37	77.59±1.34	73.96±4.69	73.81±3.15	70.97±1.91	72.99±1.49	75.41±1.99	69.16±0.58
LIN (%)		48.34±2.04^b^	57.21±4.84^a^	59.28±3.49^a^	56.68±5.73^a^	49.78±3.55^b^	40.67±3.48^b^	46.89±4.49^a^	49.69±2.97^a^	47.71±0.62^a^	41.83±1.61^b^
WOB (%)		56.21±4.94^b^	68.07±2.99^a^	66.76±1.64^a^	57.58±0.04^b^	59.47±3.19^b^	52.79±4.08^b^	60.59±1.16^a^	63.63±5.99^a^	54.74±2.46^b^	54.81±1.92^b^

Values are expressed as mean±standard error.

NMN, β-Nicotinamide mononucleotide; VCL, curvilinear velocity; VSL, straight-line velocity; VAP, average path velocity; BCF, beat-cross frequency; ALH, lateral head; STR, straightness (VSL/VAP); LIN, linearity (VSL/VCL); WOB, wobble (VAP/VCL).

a–d Different letters within the same row at the same time indicate significant differences (p<0.05).

## SUPPLEMENTARY MATERIAL

**Supplementary Figure S1.** Effect of different concentrations of NMN on sperm ROS after storage at 4 °C. Flow cytometer peak for ROS. Black, green, blue, yellow, and orange represent 0, 30, 60, 90, 120 μM, respectively.

**Supplementary Figure S2.** Different concentrations of nicotinamide mononucleotide on the expression of proteins (Bax, Bcl-2, Cleaved-Caspase3 and Cleaved-Caspase9) in ram sperm stored at 4°C.

**Supplementary Table S1.** Sperm motility parameters were analyzed by CASA
