# Supplementary material for: β-Nicotinamide mononucleotide improves chilled ram sperm quality in vitro by reducing oxidative stress damage
Source: Anim Biosci. 2024 Apr 1;37(5):852–61. doi: 10.5713/ab.23.0379 (PMC11065721; doi:10.5713/ab.23.0379)
Supplement: Supplementary file 1 [file ab-23-0379-Supplementary-Fig-1.pdf]

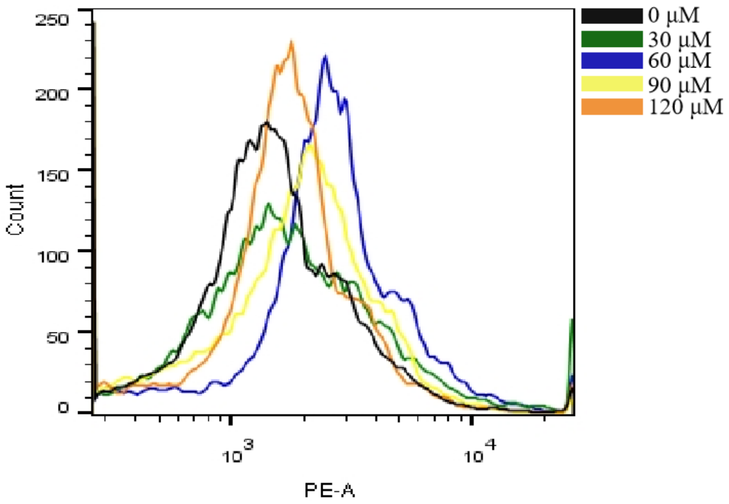

Supplementary Figure 1. Effect of different concentrations of NMN on sperm ROS after storage at 4 °C. Flow cytometer peak for ROS. Black, green, blue, yellow, and orange represent 0, 30, 60, 90, 120 µM, respectively.

.
