# Supplementary material for: β-Nicotinamide mononucleotide improves chilled ram sperm quality in vitro by reducing oxidative stress damage
Source: Anim Biosci. 2024 Apr 1;37(5):852–61. doi: 10.5713/ab.23.0379 (PMC11065721; doi:10.5713/ab.23.0379)
Supplement: Supplementary file 2 [file ab-23-0379-Supplementary-Fig-2.pdf]

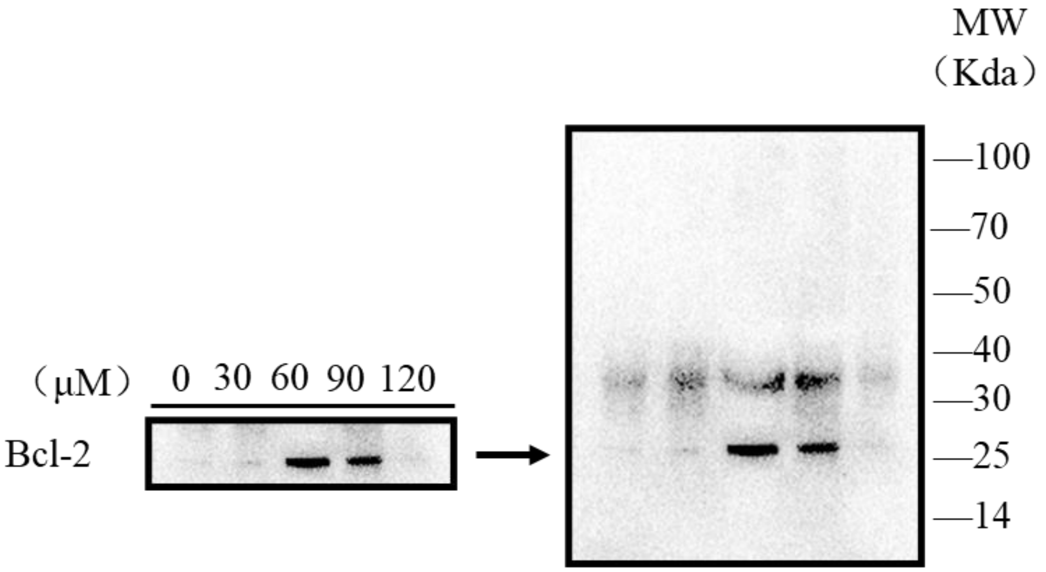
MW
（Kda）
—100
—70
—50
—40
—30
—25
—14
（μM） 0 30 60 90 120
Bcl-2

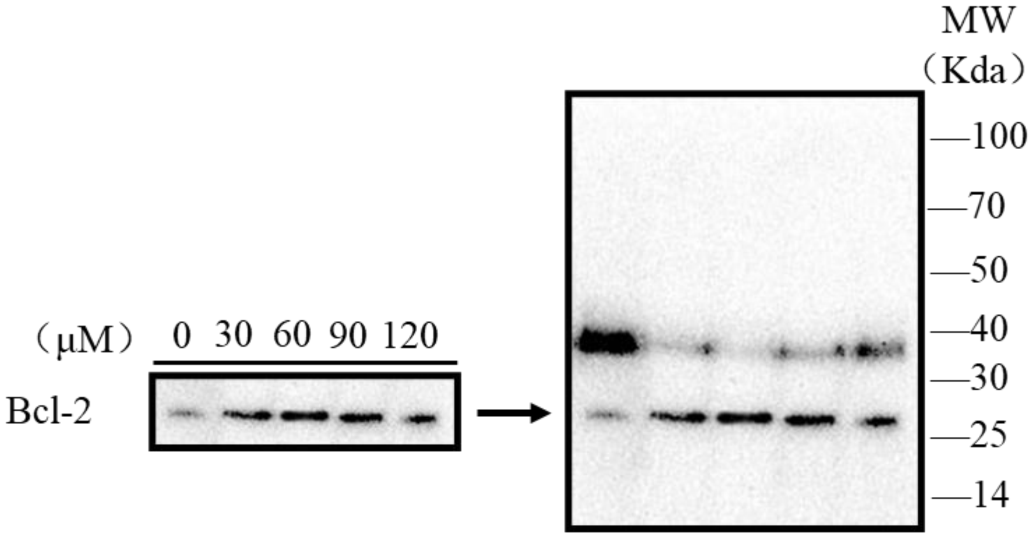
MW
（Kda）
—100
—70
—50
—40
—30
—25
—14
（μM） 0 30 60 90 120
Bcl-2

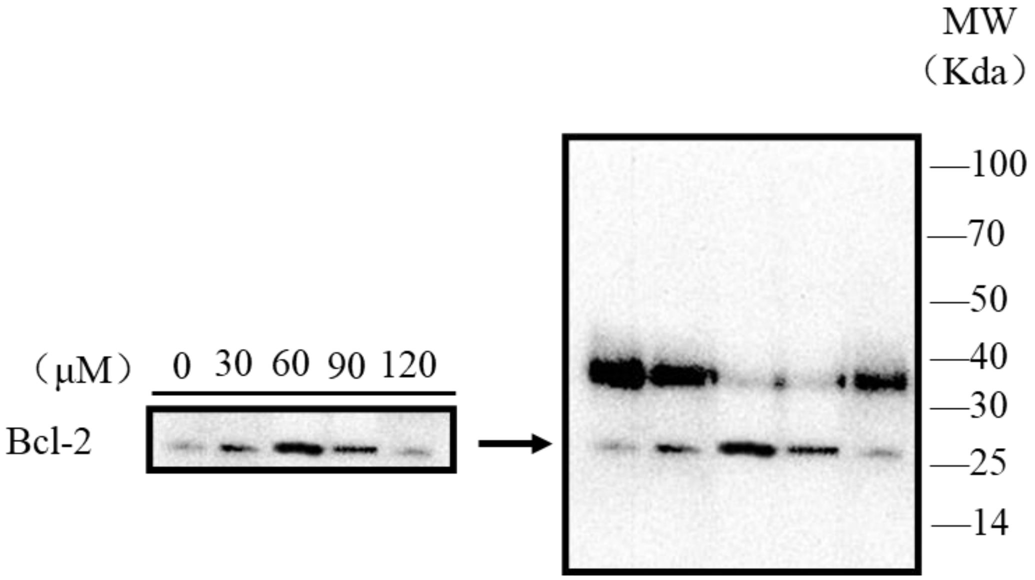
MW
（Kda）
—100
—70
—50
—40
—30
—25
—14
（μM） 0 30 60 90 120
Bcl-2

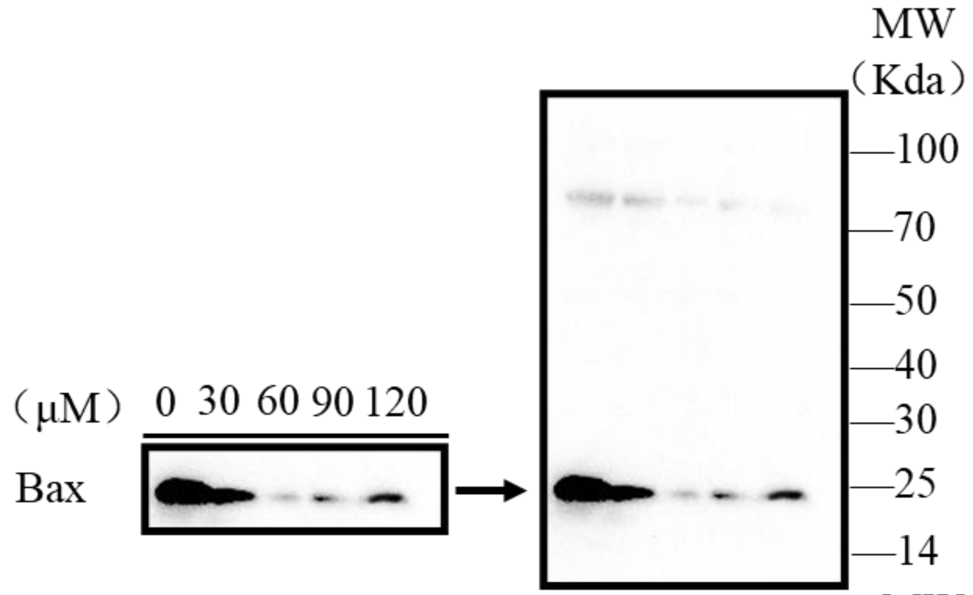
MW
（Kda）
100
70
50
40
30
25
14
（μM） 0 30 60 90 120
Bax

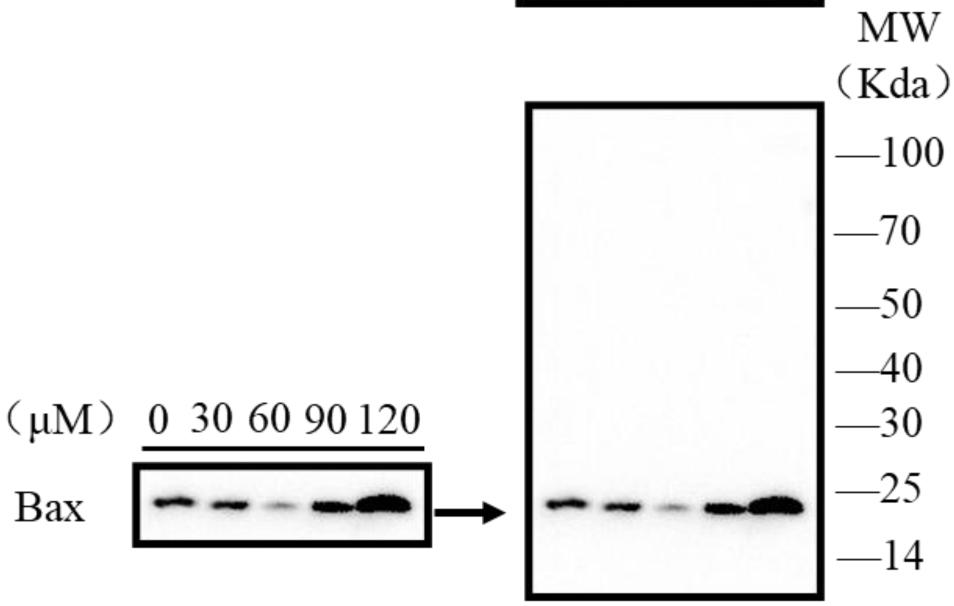
MW
（Kda）
100
70
50
40
30
25
14
（μM） 0 30 60 90 120
Bax

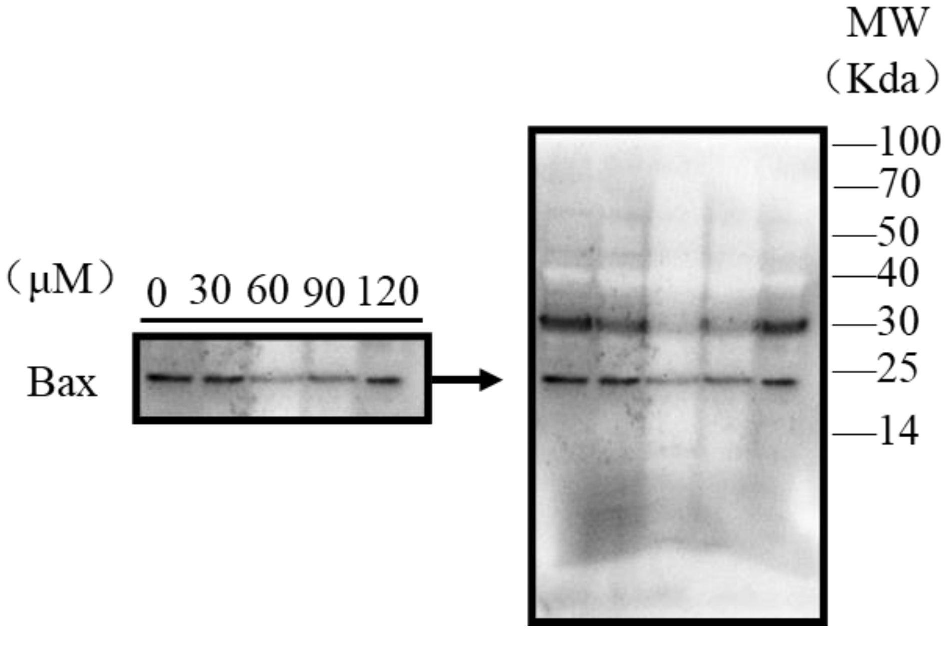
MW
（Kda）
100
70
50
40
30
25
14
（μM） 0 30 60 90 120
Bax

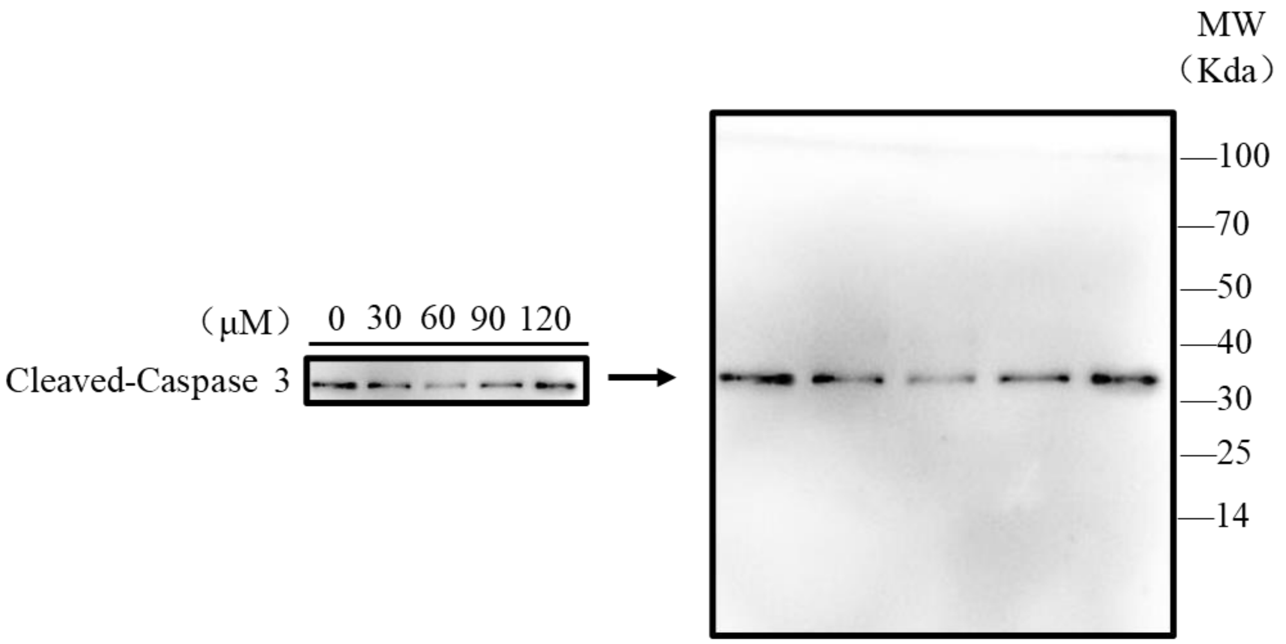
MW
（Kda）
（μM） 0 30 60 90 120
Cleaved-Caspase 3
100
70
50
40
30
25
14

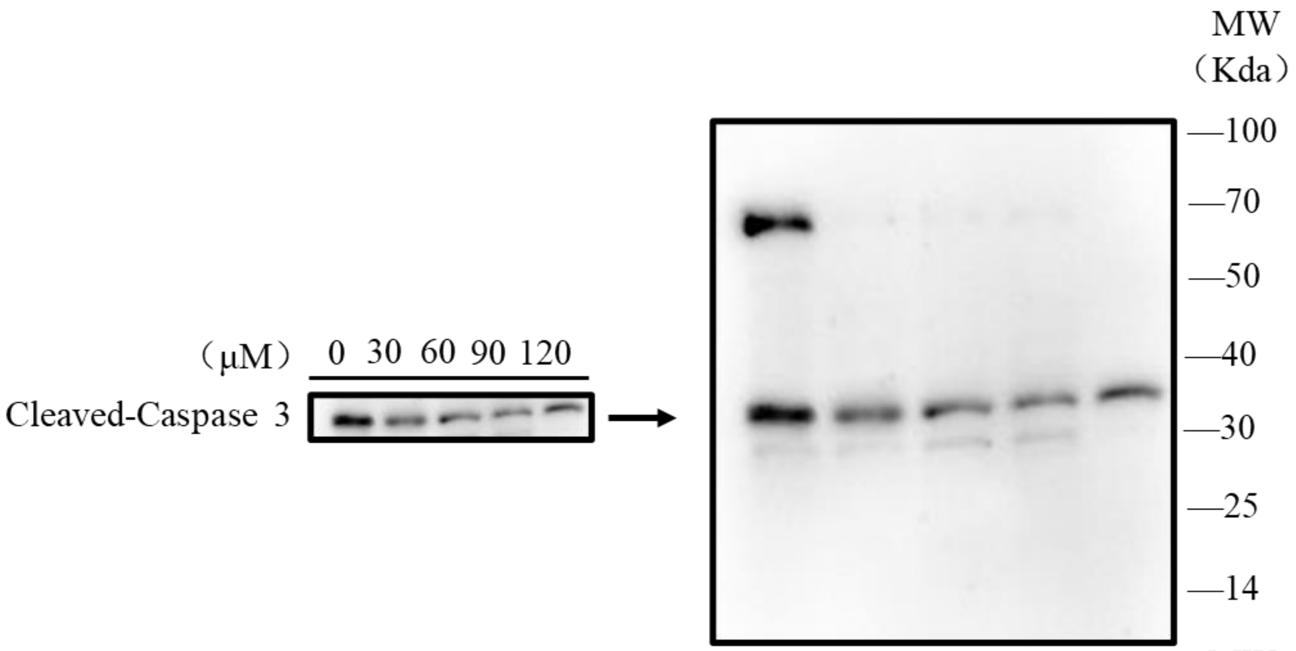
MW
（Kda）
（μM） 0 30 60 90 120
Cleaved-Caspase 3
100
70
50
40
30
25
14

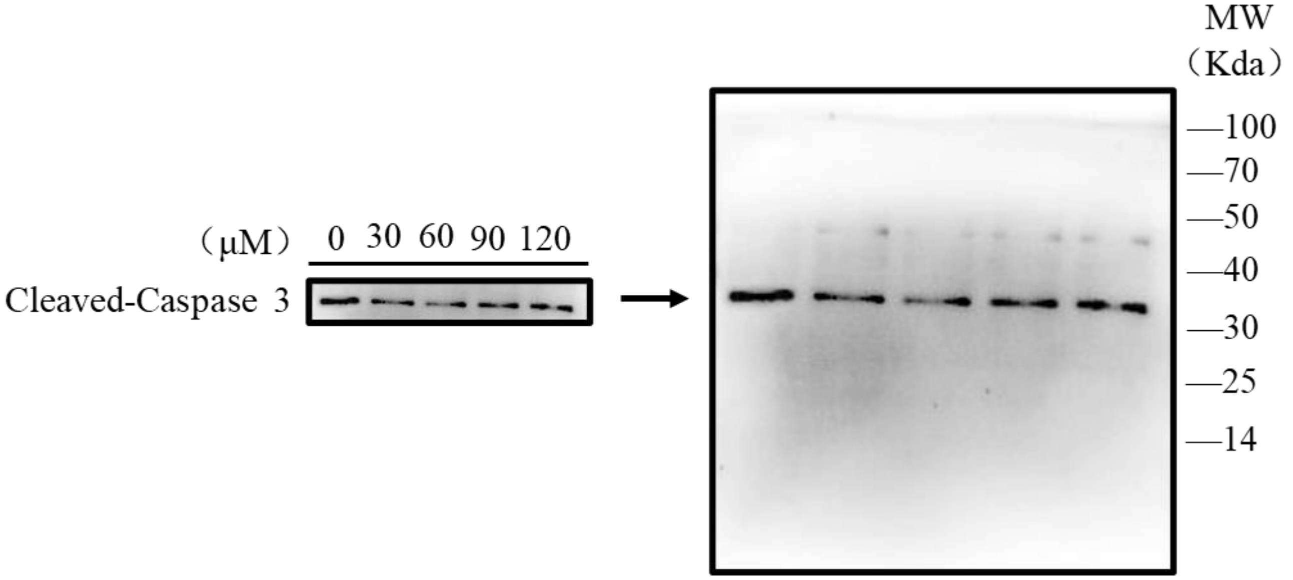
MW
（Kda）
（μM） 0 30 60 90 120
Cleaved-Caspase 3
100
70
50
40
30
25
14

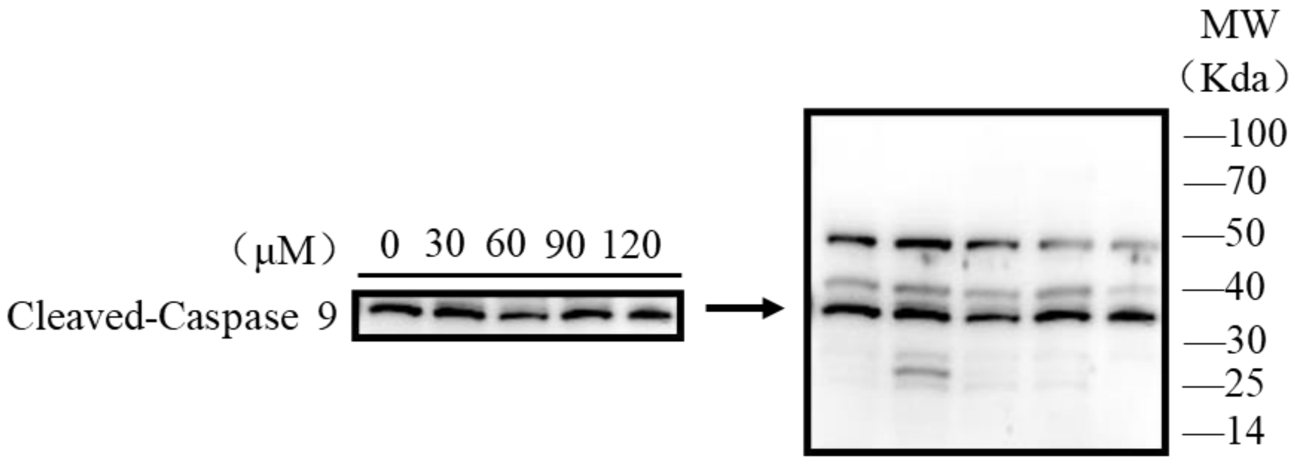
MW
（Kda）
（μM） 0 30 60 90 120
Cleaved-Caspase 9
100
70
50
40
30
25
14

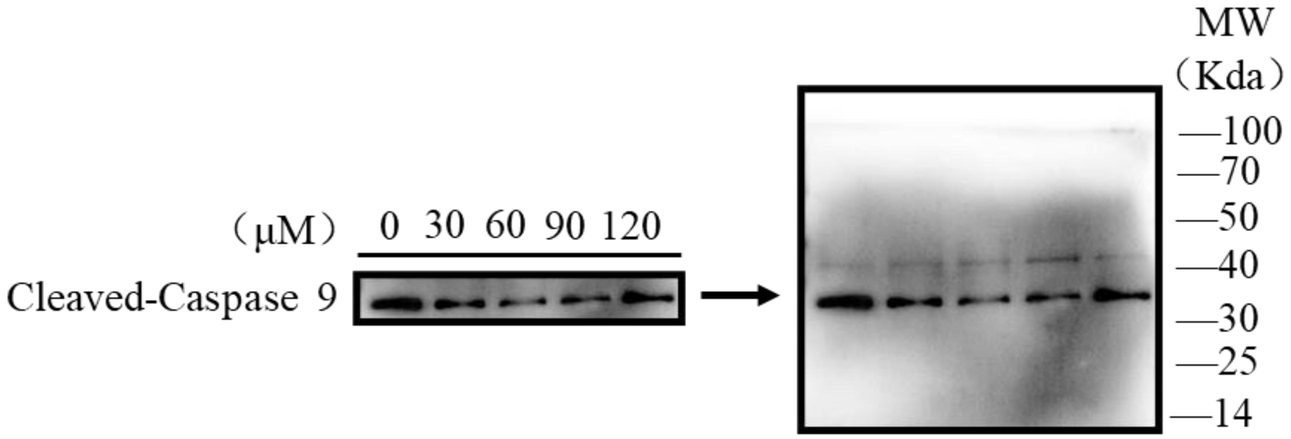
MW
（Kda）
（μM） 0 30 60 90 120
Cleaved-Caspase 9
100
70
50
40
30
25
14

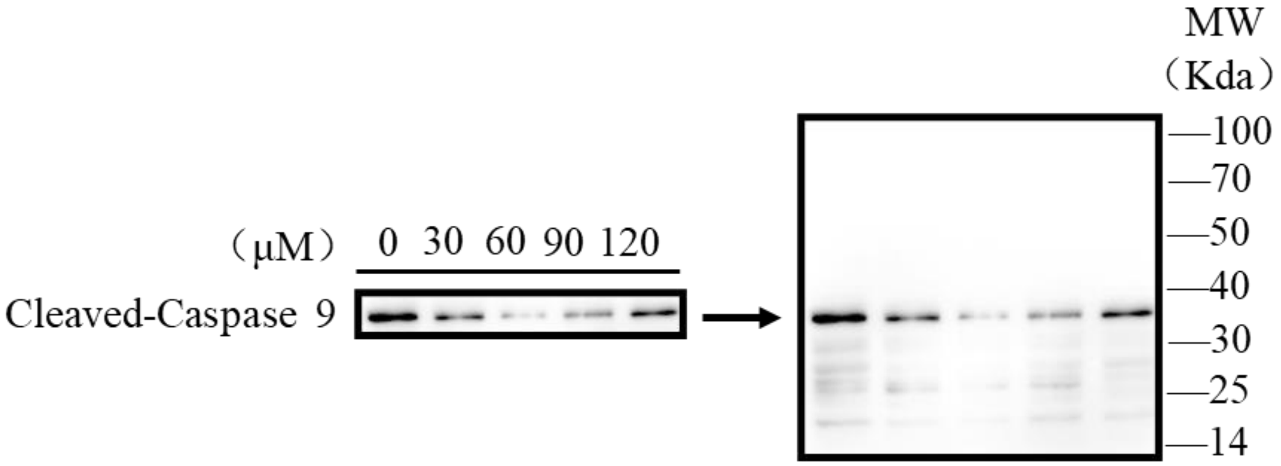
MW
（Kda）
（μM） 0 30 60 90 120
Cleaved-Caspase 9
100
70
50
40
30
25
14

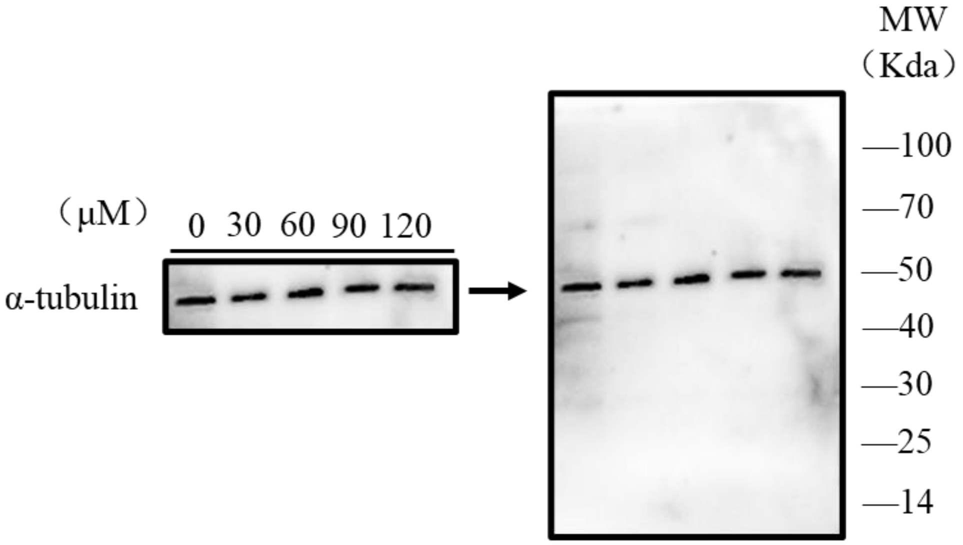

Supplementary Figure 2. Different concentrations of nicotinamide mononucleotide on the expression of proteins (Bax, Bcl-2, Cleaved-Caspase3 and Cleaved-Caspase9) in ram sperm stored at 4℃.
