# Supplementary material for: β-Nicotinamide mononucleotide improves chilled ram sperm quality in vitro by reducing oxidative stress damage
Source: Anim Biosci. 2024 Apr 1;37(5):852–61. doi: 10.5713/ab.23.0379 (PMC11065721; doi:10.5713/ab.23.0379)
Supplement: Supplementary file 3 [file ab-23-0379-Supplementary-Table-1.pdf]

Supplementary Table1. Sperm motility parameters were analyzed by CASA

Time	0 h				
Sperm parameters	0 μM	30 μM	60 μM	90 μM	120 μM
Total motility (%)	81.66±1.91	83.21±3.06	83.03±4.94	84.54±2.41	85.11±3.52
Progressive motility (%)	63.66±3.19	64.90±2.98	65.63±3.69	64.82±3.04	65.98±3.30
VCL (μm/s)	84.94±3.43	83.24±4.06	84.20±6.80	84.16±6.57	82.93±5.16
VSL (μm/s)	64.63±3.13	64.69±3.18	66.84±2.35	65.65±2.31	64.60±3.08
VAP (μm/s)	66.17±3.56	65.20±5.32	71.00±4.62	70.25±5.32	70.83±6.83
BCF (Hz)	7.95±0.31	8.12±0.28	8.16±0.88	8.73±1.14	8.11±0.55
ALH (μm)	3.86±0.25	4.19±0.67	5.26±0.50	4.82±0.17	4.95±0.15
STR (%)	85.26±2.09	84.82±4.12	84.57±4.71	83.41±5.45	84.02±5.72
LIN (%)	69.38±2.05	67.62±3.76	70.24±5.72	68.10±5.06	69.48±4.52
WOB (%)	75.14±4.16	73.20±4.69	76.79±3.48	75.38±3.82	76.17±3.31

(Values are expressed as mean ± standard deviation. Different letters within column indicate significant difference ($P < 0.05$). VCL, curvilinear velocity; VSL, straight-line velocity; VAP, average path velocity; BCF, beat-cross frequency; ALH, lateral head; STR, straightness (VSL/VAP); LIN, linearity (VSL/VCL); WOB, wobble (VAP/VCL).
